# Supplementary material for: Fluid Ferroelectric Filaments
Source: Adv Sci (Weinh). 2023 Dec 21;11(9):2305950. doi: 10.1002/advs.202305950 (PMC10916631; doi:10.1002/advs.202305950)
Supplement: Supplementary file 1 — Supporting Information [file ADVS-11-2305950-s004.pdf]

ADVANCED SCIENCE
Open Access

Supporting Information

for *Adv. Sci.*, DOI 10.1002/advs.202305950

Fluid Ferroelectric Filaments

*Marcell T. Máthé, Kelum Perera, Ágnes Buka, Péter Salamon and Antal Jákli**

Supporting Information

Fluid ferroelectric filaments

Marcell Tibor Máthé[1,2], Kelum Perera[3], Ágnes Buka[1], Péter Salamon[1], Antal Jákli[3,4,*]

[1]Institute for Solid State Physics and Optics, Wigner Research Centre for Physics, P.O. Box 49, Budapest H-1525, Hungary

[2]Eötvös Loránd University, P.O. Box 32, H-1518 Budapest, Hungary

[3]Department of Physics, Kent State University, Kent, Ohio 44242, USA

[4]Materials Sciences Graduate Program and Advanced Materials and Liquid Crystal Institute, Kent State University, Kent, Ohio 44242, USA

*: Author for correspondence: ajakli@kent.edu

Video 1: Shows extruding and then pushing back an FNLC 919 filament from a sessile drop using a micropipette. The video is in real time.

Video 2: Shows the pulling of an FNLC 919 filament in presence of $U = 20\ V$ DC voltage applied between $160\ \mu m$ diameter wires. At the end it also shows the rupture of the filament when it reached the maximum voltage until it was stable.

Video 3: Shows transversal oscillation of a $L \approx 3\ mm$ thread under $U = 50\ V$ and $f = 0.1\ Hz$ square wave voltage.
